# The procedural success of primary angioplasty in a tertiary care center in Pakistan

**DOI:** 10.12669/pjms.40.11.9954

**Published:** 2024-12

**Authors:** Abdul Wajid Khan Faisal, Khurshid Ali, Zohaib Sadiq, Zameer ul Asar, Waqas Latif, Madiha Iqbal

**Affiliations:** 1 Abdul Wajid Khan Faisal, FCPS (Medicine); FCPS (Cardiology) Professor of Cardiology, Department of Cardiology, Punjab Institute of Cardiology, Lahore, Pakistan; 2 Khurshid Ali, FCPS Fellowship in Interventional Cardiology, Cardiologist, DHQ Hospital Batkhela Malakand, Pakistan; 3 Zohaib Sadiq, FCPS Senior Registrar Cardiology, Punjab Institute of Cardiology, Lahore, Pakistan; 4 Zameer ul Asar, FCPS (Cardiology) Assistant Professor of Cardiology, King Edward Medical University, Lahore, Pakistan; 5 Waqas Latif, FCPS (Cardiology) Assistant Professor of Cardiology, King Edward Medical University, Lahore, Pakistan; 6 Madiha Iqbal, FCPS (Cardiology) Assistant Professor of Cardiology, King Edward Medical University, Lahore, Pakistan

**Keywords:** Primary angioplasty, TIMI flow, MBG, Procedural success

## Abstract

**Objective:**

This study was conducted to evaluate the procedural success of primary angioplasty in terms of TIMI flow and myocardial blush grade (MBG).

**Methods:**

We used cross-sectional population data of the patients who underwent primary PCI in Emergency Angiography department of Punjab Institute of Cardiology, Lahore from January 2021 to September 2021. TIMI flow and myocardial blush grades (MBG) were assessed before and after the procedure. All PCIs in which there was improvement in blood flow in infarct related artery of ≥2 grades in TIMI flow and/or in MBG were considered successful.

**Results:**

A total of 106 patients were enrolled, 91 patients were male and 15 patients were female with the age range from 22 to 75 (48.7 ± 11.8) years. TIMI flow and MBG was improved ≥ 2 grades in 69 and 58 patients respectively. Improvement in TIMI flow was noted similar in all three vessels. Overall 65% patients showed improvement in TIMI flow and 55% in MBG. Onset of chest pain to balloon time was the most important parameter, shorter the time better the result. The patients in whom MBG was improved, the time was 234.26 ± 156.06 vs 323.59 ± 252.64 minutes.

**Conclusion:**

Primary angioplasty is quite effective procedure for the restoration of blood flow in acute ST elevation myocardial infarction but still it is far away from the ideal one. Although most patients benefit from primary PCI, a significant minority of individuals do not, particularly if they present late. What could be the ideal therapy, the answer is still to be answered.

## INTRODUCTION

Primary angioplasty also known as primary PCI is performed world over as a successful intervention for ST elevation myocardial infarction (STEMI).^1^ The ultimate goal is restoration of blood flow to the ischemic myocytes. The blood flow to the myocardium can be assessed angiographically by myocardial blush grade.

Higher the myocardial blush grade, better the outcome.^2^ The occluded coronary arteries in acute STEMI can be opened up in up to 96% of cases by primary percutaneous intervention (primary PCI).^3^ Even after achieving the epicardial coronary vessel patency, primary PCI may fail to restore optimal myocardial reperfusion within the myocardial tissue in patients with STEMI. The lack of reperfusion at the microvascular level is a condition known as no-reflow (NR).^4^ No reflow has been described in up to 60% of STEMI patients with optimal coronary vessel reperfusion. The possible mechanisms of this phenomenon include endothelial dysfunction, microvascular dysfunction, spasm, embolization, and reperfusion injury.^4^

TIMI flow is an important parameter to assess the flow to ischemic myocardium before and after the procedure in acute ST segment elevation myocardial infarction. Thrombus burden, total stent length, low systolic blood pressure and TIMI flow ≤ 1 at presentation are predictors of poor outcome. TIMI flow ≤ 2 after the procedure is associated with adverse outcome.^5^

Myocardial blush grade is the final goal to ascertain the perfusion of myocytes. In order to determine whether primary PCI is accomplishing the intended purpose or not is still debatable. What should be the preferred approach to preserve the myocardium in order to ensure appropriate reperfusion? needs to be studied.

## METHODS

One hundred six patients in whom primary angioplasty was performed were enrolled in the study which was conducted from January 2021 to September 2021 at the angiography department of the institution. Detailed consent was taken from the attendants before the procedure. Known cases of ischemic heart disease that had prior history of MI, PCI or CABG were excluded. All patients in whom primary PCI could not be performed either due to unsuitable anatomy for PCI or attempted /aborted PCIs were excluded. TIMI flow and myocardial blush grades (MBG) were assessed before and after the procedure.

### Ethical Approvals:

The study was approved by the ethical review committee of Punjab Institute of Cardiology, Lahore (Ref. No. RTPGME-Research-148 dated: December 15, 2020).

All PCIs in which there was improvement in blood flow in infarct related of ≥2 grades in TIMI flow and/or in MBG were considered successful. TIMI flow and MBG was assessed by the operator as well as by an expert and grading was done by the mutual consensus. The grading was done according to the standard definitions. Fisher exact test, chi square and t test were used for significance. Data was analyzed on SPSS 16, p-value of less than 0.05 was considered significant.

## RESULTS

A total of 106 patients were enrolled, 91(85.8%) patients were male and 15(14.2%) patients were female with the age range from 22 to 75 (48.7 ± 11.8) years. The left anterior descending artery (LAD) was the most common infarct-related artery 56(52.8%) followed by RCA 36(34%) and LCX 14(13.2%), (Table-I). Smoking 45.3% (48 patients) was the major risk factor in the study population. Diabetics were 26.4% (28 patients) and 34% (36) patients were hypertensive. GpIIb/IIIa inhibitors were used in addition to DAPT in 68(64.2%) patients during the procedure. As expected most of the patients had TIMI flow and MBG of grade 0 before the procedure i.e., 60.4% (64) and 80.2% (85) respectively.

**Table-I:** Baseline characteristics of the study population.

Age	48.66±11.7 years (mean) 22-75 years	
*Characteristic*	*Categories*	*Frequency (n)*
Gender	Male	91 (85.8%)
	Female	15 (14.2%)
Vessel	LAD	56 (52.8%)
	LCX	14 (13.2%)
	RCA	36 (34.0%)
Risk factors	Diabetic	28 (26.4%)
	Hypertensive	36 (34.0%)
	Smoking	48 (45.3%)
Platelet inhibitor	GpIIb/IIIa	68 (64.2%)
Pre TIMI	0	64 (60.4%)
	1	16 (15.1%)
	2	18 (17.0%)
	3	08 (07.5%)
Pre MBG	0	85 (80.2%)
	1	07 (06.6%)
	2	11 (10.4%)
	3	03 (02.8%)

After the procedure, TIMI flow was improved by ≥ 2 grades in 69 (65%) patients and MBG was improved by ≥ 2 grades in 58 (54.7%) patients. ECG changes settled in 84 (79.2%) patients and chest pain was resolved in 100 (94.3%) patients. In two patients (1.9%) the TIMI flow actually decreased from the pre-procedure and in one patient (0.9%) MBG decreased by one grade. Overall there was a significant improvement in TIMI flow and MBG (p value.001) after the procedure (Table-II). Improvement in TIMI flow was noted similar in all three vessels i.e. 66.1% in LAD, 64.3% in LCx, and 63.9% in RCA (p-value 0.975) (Table-III).

**Table-II:** Post PCI outcomes.

Characteristic	Categories		n (percentage)
Post TIMI	0		2 (1.9%)
	1		5 (4.7%)
	2		38 (35.8%)
	3		61 (57.5%)
Post MBG	0		11 (10.4%)
	1		20 (18.9%)
	2		33 (31.1%)
	3		42 (39.6%)
ECG	Complete recovery		84(79.2%)
	Partial		4(3.8%)
	Q wave		5(4.7%)
	No improvement		8(7.5%)
	Unknown		5 (4.7%)
Chest Pain (CP) resolution	Complete		100(94.3%)
	Partial		2(1.9%)
	No		3(2.8%)
	Unknown		1 (0.9%)
Post PCI Change in TIMI score	- 1		2(1.9%)
	0		13(12.3%)
	1		22(20.8%)
	2		39(36.8%)
	3		30(28.3%)
Post PCI Change in MBG score	- 1		1(0.9%)
	0		17(16%)
	1		30(28.3%)
	2		29(27.4%)
	3		29(27.4%)
	Pre	Post	p-value
MBG	0.36±0.78	2.0±1.01	0.001
TIMI	0.72±1.00	2.49±0.68	0.001

**Table-III:** Post PCI improvement in TIMI flow (>2 grades) in different vessels.

Vessels	Post PCI TIMI flow (>2 grades)		P-value

	Yes	No	
LAD	37(66.1%)	19(33.9%)	0.975
LCx	9(64.3%)	5(35.7%)	
RCA	23(63.9%)	13(36.1%)	
Total	69(65.1%)	37(34.9%)	

A higher improvement rate in MBG was observed in patients with disease in RCA (72.2%) followed by LCX 64.3% and LAD 41.1% with a significant p-value of 0.010 (Table-IV). Overall 69(65%) patients showed improvement in TIMI flow and 58(55%) in MBG. The female patients 12(80 %) showed marked improvement in both TIMI flow and MBG especially the MBG grades with a statistically significant p-value of 0.034 as compared to the male patients (Table-V).

**Table-IV:** Post PCI improvement in MBG (>2 grades) in different vessels.

Vessels	MBG (>2 grades)		P-value

	Yes	No	
LAD	23(41.1%)	33(58.9%)	0.010
LCx	9(64.3%)	5(35.7%)	
RCA	26(72.2%)	10(27.8%)	
Total	58(54.7%)	48(45.3%)	

**Table-V:** Characteristics of the patients having successful Vs patients not having successful PCI.

Characteristics		Increased TIMI (≥ 2) (n=69)	Increase in TIMI (< 2) (n=37)	P-Value	Increased MBG (≥ 2) (n=58)	Increase in MBG (< 2) (n=48)	P-value
Gender	Male	57(62.6%)	34(37.4%)	0.191	46(50.5%)	45(49.5%)	0.034
	Female	12(80%)	3(20%)		12(80%)	3(20%)
Age		49.28±11.7	47.51±11.7	0.465	49.03±11.16	47.51±12.49	0.431
CP BT (min)		278.23±191.42	270.0±243.42	0.853	234.26±156.06	323.59±252.6	0.033
DM		21(75%)	7(25%)	0.439	18(64.3%)	10(35.7%)	0.456
HTN		28(77.7%)	8(22.3%)	0.145	19(52.7%)	17(47.3%)	0.941
Smoker		27(56.2%)	21(43.7%)	0.183	22(45.8%)	26(54.2%)	0.246
Gpllb/Illa		48(70.6%)	20(29.4%)	0.193	40(58.8%)	28(41.2%)	0.334
CP Resolution	Complete	67(63.2%)	33(31.1%)	0.313	57(53.8%)	43(40.6%)	0.222
	Partial	1(0.94%)	1(0.94%)		0	2(1.88%)	
	No	1(0.94%)	2(1.88%)		1(0.94%)	2(1.88%)	
ECG	Complete	55(51.9%)	29(27.3%)	0.622	45(42.45%)	39(36.8%)	0.182
	Partial	2(1.88%)	2(1.88%)		1(0.94%)	3(2.82%)	
	Q wave	4(3.77%)	1(0.94%)		5(4.7%)	0	
	No	6(7.54%)	2(1.88%)		5(4.7%)	3(2.82%)	

CP BT: Chest pain to balloon time

MBG was improved in those patients who presented early that is the time from onset of chest pain to the balloon was 234.26±156.06 minutes as compared to 323.59±252.6 minutes in patients who did not show improvement [p-value 0.033]. GpIIb/IIIa inhibitors were used in 68(64%) patients, around 48(70.6%) of the patient in whom GpIIb/IIIa were used showed improvement in TIMI flow and 40(58.8%) patients had improved MBG of ≥2 grades.

## DISCUSSION

In our study, 106 patients with primary PCI were included. TIMI flow and MBG improved by ≥ 2 grades in 69 and 58 patients, respectively. The improvement in TIMI flow was similar in all three vessels. Overall, 65% of patients showed improvements in TIMI flow and 55% in MBG. The onset of chest pain to balloon time was the most crucial metric; the shorter the time, the better the outcome. The time for patients with improved MBG was 234.26 + 156.06 vs 323.59 + 252.64 minutes.

Primary PCI is done as a Class I treatment for ST segment elevation acute myocardial infarction.^6^ Primary PCI shows better results than fibrinolytic therapy but it has own hazards and complications.^7^ Moreover, not all centers have the facility of primary PCI and the cost is also an issue specially in third world countries. So we must critically analyze the results and then plan the future strategies to deal with acute MI. The therapy must be cheap and widely available and with lesser expertise it can be implemented. We planned this study in our setup to actually analyze the benefit this therapy is providing to our patients.

Improvement in TIMI flow (TIMI flow 3) in primary PCI is 78-90% and even more than that.^5^,^8^ Sharma et al in their study showed myocardial blush grade of 2-3 in 72% of patients where thrombectomy was used and 70 % where thrombectomy was not used.^9^ In this study, 55% patients had MBG of 2-3 because the patients who presented in mean time of less than four hours, while those patients who presented late were not benefited. In study by Sharma et al, 85% of patients presented early, that made the difference in the results.^9^

Caixeta et al showed TIMI three improvement in 87% patients.^10^ Whereas, in a study by Shaaban et al TIMI 3 flow and MBG Grade-3 was achieved in 75% patients.^11^ Elakabawi et al.^5^ showed that TIMI 3 flow achieved in 77% patients. In another study by Duyuler PT et al.^12^ MBG Grade-3 was achieved in 44% patients only. The underlying mechanisms of reduced blood flow to myocardium (lower MBG) are microvascular obstruction secondary to ischemia, distal embolization and reperfusion injury.^13^

Brickemeyer et al.^14^ showed that female gender had adverse outcome after primary PCI. In a study by Yasmin et al.^15^ from a developing country showed no difference in outcome in male and female patients after primary PCI. Chou et al.^16^ and Krishnamurthy et al.^17^ also had similar results but contrastingly in our study female patients though lesser in number had better procedural results, in hospital and the long term outcome was not studied by us. RCA involvement had better results than LAD but the data regarding this fact is scarce in literature, what might be the reason is not known. Entezarjou et al. showed in their study that primary PCI of RCA has better outcomes in terms of death, heart failure and stroke as compared to LAD.^18^

Smokers had lesser grade of TIMI flow and MBG but the P-value was insignificant. Boulos et al. showed post PPCI improvement in MBG ≤2 was 62.12% in smokers and 75.7% in non-smokers. Doppler flow measurements in the descending left coronary artery were performed. In the early flow, smokers and non-smokers have similar diastolic filling times (11.9), while smokers have double the sluggish systolic filling time (6.48 ± 16.4) compared to non-smokers (3.26 ± 1.53).^19^

What could be the ideal management of acute MI, fibrinolytic, primary PCI or fibrinolytic followed by PCI within 3-24 hours. Data from stream and fast AMI^20^ showed five years survival rate of 88% in fibrinolytic group and 84% in primary PCI group. So, the debate is open for preferred strategy specially if patient presenting within three hours of acute MI, the fibrinolytic is very effective and Tenecteplase has the superiority.

### Novelty of the study:

Primary PCI is performed world over as a standard of care in STEMI patients. Our study showed that around 35% of the patients does not get 100% benefit. So we must think a way forward to have a better management of STEMI patients to provide maximum benefit to almost all patients.

### Limitations:

This is a single-center study with a small sample size, it is not possible to generalize the findings. To validate the results and offer recommendations for the future, a sizable multi-center trial is required.

## CONCLUSION

Primary angioplasty is quite effective procedure for the restoration of blood flow in acute ST elevation myocardial infarction but still it is far away from the ideal one. Although a significant majority of patients get benefit from primary PCI, but still a mentionable number of patients do not benefit fully from it, especially if they present late. What could be the ideal therapy, the answer is still to be answered. Howsever, fibrinolytic therapy followed by angioplasty may show promising results in future studies.

### Authors Contribution:

**AWKF:** Planned the study, collected the data, manuscript writing. Accountable for the accuracy and integrity of the work.

**KA** and **ZS:** Helped in data collection, literature search and critical review.

**ZA:** Prepared research protocol, designed study proforma and literature search.

All authors have read and approved the final manuscript.
